# Discussions before the Kentucky State Dental Association

**Published:** 1878-08

**Authors:** 


### Proceedings of Societies.

### DISCUSSIONS BEFORE THE KENTUCKY STATE DENTAL ASSOCIATION.

*Continued from page 298.*

Therapeutics was the next subject assigned for discussion.

Dr. McMillan read an interesting paper upon the subject. At its close Dr. Taft said: The subject of neuralgia is one with which the dentist very frequently comes in contact, and the cause is commonly obscure; still the cause is not so difficult to ascertain as to remove. Undoubtedly the great majority of cases will be found to come from impingement somewhere. The hypertrophy of the roots of the teeth often produces neuralgia. There is often systemic treatment employed that does not accomplish much in effecting a cure of neuralgia—a great deal of fruitless medication in the treatment of this affection. Exsection of the nerves has been practiced with decided success. I have known instances where exsection had been performed the second time, the nerves having united and neuralgia returned after the first exsection. This might call up the question, do the nerves of the pulps of living teeth ever unite after they have been removed and replaced? It has not very often occurred, but it does sometimes. I know a case of neuralgia where a gentleman in Cincinnati has suffered for nearly ten years, and he has sought relief from all sources without finding it, though he is some better now than formerly. I know a gentleman who some years ago suffered intensely with facial neuralgia, and he had about four lines of a nerve removed, and has had no return of the difficulty since, though for months before he had been unable to do anything on account of the excruciating pain.

Dr. Roudebush said that a case came under his observation once, where the neuralgia had extended from the face down into the arm. He found no decayed teeth, and applied to Dr. Dawson who excised the medial nerve of the arm, taking out about two inches which seemed to relieve the patient for a time, but he was still troubled at times with facial neuralgia. He then concluded that constitutional treatment was the only hope, and commenced with iodide of potassium, which helped him somewhat. Then changed to bromide of potassa with better results, but when the medicines were discontinued the disease returned again. That syphilis was a frequent cause of neuralgia had been discovered beyond a doubt by physicians who examined into the causes.

Dr. Sage—I was informed by Dr. Blackbnrn, of Cincinnati, an eminent physician now dead, that he had once or twice performed exsection of the medial nerve of the upper arm, taking out about four inches, which was afterward restored, and since that the arm had been in an almost paralyzed condition. I have often wondered whether the pulps of the teeth of vigorous people could not be restored, but I confess that I am not able to give a reason for any such belief. Cited the case of an uncle of his who had suffered exsection of the nerve, and had every tooth in his mouth extracted without any relief, the disease remaining a secret to the day of his death.

Garretson's oral surgery contained a most interesting essay on neuralgia which he commended to the attention of the members of the Association. Subject passed.

Dr. A. W. Smith, the President, then addressed the Association stating that when he became a member he received a diploma signed by Geo. W. Priest as president, that he returned the diploma to the Association as he had no further use for it; that Dr. Priest had violated his honor and his membership by quack practices; that his course had been of a character that had brought dishonor on any diploma bearing his name, and he desired a diploma signed by some of the honorable members. He then moved that all the diplomas issued, bearing the name of G. W. Priest as President or Secretary, be rescinded and new diplomas issued. Carried. On motion of Dr. Goddard, the President was authorized to procure as many diplomas as might be required to take the place of those signed by Dr. Priest and other members. Carried. Adjourned.

THURSDAY MORNING SESSION.

The first subject taken up at this session was Surgery. Dr. Edwards read a paper upon treatment of exposed pulps.

Dr. Taft—A glance at the tissue under discussion will indicate something to us of the nature of the appropriate treatment. When it becomes exposed it must be restored to its original condition. That this is possible there is abundant evidence to prove; that it fails sometimes can only be expected. Pulps sometimes become devitalized when not exposed at all, and occasionally when there is no decay; of course, such can not be preserved, but in a large proportion of cases it is possible to save exposed pulps, and it is an erroneous idea to suppose that the pulps of teeth are of very little account after the complete development of the teeth, and might as well be destroyed. Every one of experience and observation knows that a devitalized tooth is not as good as the living one, that it is susceptible to disintegrating influences in a far more marked degree, and that the living tooth will not as readily decay as the devitalized tooth. I regard it as a very important matter to preserve the living teeth as long as possible. When a diseased pulp is presented, it should first be restored to its normal condition, and then protected by something which will do that without pressure, so that some plastic material is probably better than any thing else. And it must be something that will not be decomposed. It should occupy all the space and make no pressure. Oiled silk is used with success, placing os-artificial over it. He had found very little difficulty in using lacto-phosphate of lime, or even lactic acid and lime.

In regard to replanting teeth, the paper seemed to claim that the parts should all be in good healthy condition. Now by far the greater number of cases where I have seen the operation performed, it has been for chronic inflammation of the periosteum and alveolar abscess, and in nearly all the cases of that kind it was successful. Where there is considerable enlargement about the root of the tooth occasioned by an abscess and destruction of tissue, the teeth may be taken out, the debris removed, and the teeth returned and all be well. I had a case, May, 1876, where there had been a fistulous opening in the cheek for more than a year, it had been in a physicians charge for months without relief. I removed the tooth, and inferior bicuspid, cleansed off the debris from the tooth and cleansed out the socket. The tooth was then replaced and within a few days the flow ceased, and there has been no discharge whatever since, and the tooth is just as serviceable in mastication as it ever was. I have been familiar in the last three or four years with perhaps one hundred and fifty cases of replanting and don't know of more than six failures. Out of eighty cases in the Infirmary at Michigan, only one failed. Out of about thirty cases in his office practice, there had been one, two or three failures.

Dr. Thomas reports the case of a man who had the root of a cuspid tooth that was so far gone that a crown could not be set upon it. About a month before, he had extracted a cuspid tooth from a lady which he thought would just fit this cavity. The tooth had been thrown away with the debris, but he found it, and placed it in the cavity from which he had drawn the root, and within a few days it grew firmly in the socket, and several months afterwards it was examined by several dentists, among them Dr. Watling and Dr. Field, and they said you could not tell that tooth from the others in the mouth.

The President—I think this subject of the treatment of the pulp a very important one, and, as I have been making some investigations in regard to that matter, I will give them merely as experiments, hoping to receive assistance from others. It is the method of using the natural dentine as a capping for the dental pulp. I don't know that this has ever been done. for the past year I have been making some experiments in that line; I did think probably I had best wait further developments before bringing it before the Association, but if there are half a dozen or dozen who will enter into the experiments, there will be that many more results. We have in this dentine the natural tooth substance which is easily procured and pulverized. In some cases I have mixed it with lactic acid, something similar to lacto-phosphate of lime, and spread it over the pulp of the tooth. Another method is to take the dentine and sprinkle it perfectly dry over the pulp, cut a small piece of oiled silk and put over that, and then cap it with oxi-chloride of zinc. I have been experimenting with it for about a year now, and in every case except one the teeth are doing well. Whether any change takes plaec in the pulverized dentine or not, I am unable to say. I was speaking with Dr. Cassidy this morning, and he thinks not. I think if it will take up the carbonates and phosphates of lime, it would be more natural to take up the dentine than the artificial material, though I don't say that it will do that.

Dr. Peabody—It would seem to me that this powdered dentine sprinkled on the pulp, would act very similarly to secondary dentine unless it was thoroughly dissolved and taken up by the pulp. Now I would like to ask Dr. Taft if he knows of many cases where an old dry tooth from other people's mouths has been put in and grown, and been serviceable. I have heard Dr. Cutter went so far as to say that you could take a piece of ivory and whittle it down to a peg, and insert it, and it being dentine, it would be received by the system and not thrown off.

Dr. Taft—I do not believe that such a tooth would become attached; have not seen such a case, nor have I seen one who has.

Dr. Peabody—In every case of my experience the tooth has dropped down and become very sore. I would like to hear the experience of others on that point. I have heard that excision of the apex of the root will prevent the dropping down—has any one had experience as to that?

Dr. Rawles—I believe I can solve that question for Dr. Peabody. Cutting off the apex does not help the matter. I have seen a tooth drop down when nearly one-third was cut off. You don't interfere alone with the apex, but with the sides, and consequently you have then a tendency which would alone cause it to drop down, without considering the animal fluids at the apex of the root, and it would drop down sufficiently to allow the animal fluids to be swept out, and it don't matter whether you cut off a thick or thin portion so you allow it to drop down low enough for that purpose, and then give them time to pass away and the tooth will in time get back to its position. In regard to why we may not expect the formation of membrane around the tooth after it has been out some time, it seems to me it depends altogether upon the time it has been out, and whether the membrane is entirely upon the tooth or the socket. If in the socket it does not seem at all improbable that the union would take place. If it had dried up it would be kept more by mechanical agency than anything else. If the tooth is properly filled and replaced, even though it be dry throughout, it might be retained in position by its integrity alone, and the mechanical adaptation of the jaw around it. Ivory pegs put into any part of the bone of the body are disintegrated, not thrown off entirely, but just disintegrated.

Dr. Taft said he had but little faith in the mere mechanical retention of the tooth in the mouth. There would be irritation under a mechanical pressure strong enough to hold the tooth in.

Cited the case of a little girl who fell and broke off the edge of the central incisor to the gum on one side and about one-third on the other, exposing the pulp. Dr. Field, to whom the case was brought, removed the tooth, put it down to its neck in plaster, and built up a crown of gold and replaced it. Four or five months afterward he saw the tooth, and you couldn't tell it had ever been out of the mouth, and the child was using it with perfect freedom. The tooth was in plaster three hours before it was replaced, and the living parts received it kindly and united with it.

Dr. Peabody—If Dr. Rawles' theory that the tooth is held in place by mechanical means is correct, you might take a piece of lead and fit it to the cavity and obtain the same result, and it will be kept in its place. Now we all know that any dry substance, such, for instance, as a piece of leather, if placed in moisture will increase in size, and the vascular formation will become quite distinct again. Now may it not be that when these old teeth are replaced in the mouth they are softened by the moisture and the fluids of the mouth, the vessels are enlarged, and the vascular formation permits the flow of those fluids, and the tooth is not held there by mechanical means only, but by and through a living attachment. This discovery seems to indicate that there is simply an attachment, and no vitality whatever.

Dr. McMillan—I have had the same trouble with the elongation of the teeth that Dr. Peabody spoke of, and in two or three instances had to remove the tooth three or four times. In every case where I waited a sufficient time to syringe out the cavity thoroughly, I had no such trouble, and in every case where I did not thoroughly cleanse the parts, I had further difficulty, whether I shortened the apex of the root or not—that seemed to make no difference whatever. The least particle of secretion that is left gives trouble.

The next subject presented was Treatment of Children's teeth.

Dr. Kidd read a paper on the subject.

Dr. Sage said it had been his custom to retain the temporary teeth as long as possible, in order to prevent the contraction of the jaw and the space necessary for the new teeth. He had but little experience with filling temporary teeth as children would not submit patiently enough to make a successful operation. Saltpeter or common nitre he used as a palliative remedy when necessary. He was glad Dr. Kidd had placed the six year molars among the childrens teeth. When they decayed he extracted them.

Dr. Goddard—I think the best thing we can do in regard to the preservation of children's teeth is to educate the parents. The children themselves are not to blame; they are ignorant and don't know the consequences, and nineteen-twentieths of the parents don't either. They suppose the six year molar teeth are deciduous and are to be lost. If they could be educated up to know that they are not it would help the matter. It has been my habit to use files to file away the caries on children's teeth, and if the tooth aches I use something to ease the pain and let the tooth remain. I think there ought to be articles in every paper, especially every Sunday paper, referring to children's teeth. I always endeavor to save the six year molars if I possibly can. If I can save them until the child is twelve or thirteen years old I think I have accomplished much. I never want to extract these teeth until after the child is twelve years old. Children should be taught to use a soft brush as soon as they possibly can, and it should be made an imperative duty to have them washed at night.

Dr. Taft had but little faith in the publication of articles in the newspapers; people would not read them. He thought the dentist should instruct his patients personally as to this matter, and if they were ignorant he could blame himself for it. The distribution of little works on the subject would do much to educate the people up to a proper appreciation and discharge of their duty in this respect. Subject passed temporarily.

Dr. Peabody then read a paper on a new method of inserting pivot teeth.

AFTERNOON SESSION.

The Association convened at 2:30, and the discussion on the subject of the care of childrens' teeth was resumed.

The President—I was appointed on the committee to write on this subject, but being president excused me from other work according to the resolution passed by the Association. I have no children of my own that I know of, but I have a method of educating parents and children on this subject, and that is to get on familiar terms with the children of my patients—have them about you, get acquainted with them, know their names and have them come and see you frequently. In my office I have a suite of five rooms and one of them I have fitted up as a play house for the children. I purchased a large music box, paying three hundred and fifty dollars for it, and have other attractions that will draw the children. Saturdays I have set apart for the last seven years for the examination of children's teeth. I make no engagements on that day. Sometimes on that day I have thirty or more children come up to see me. I have made my office a public resort for the children, whereas the dentist's office is generally regarded as a very horrible place. Parents will frighten their children by saying, "I will have the dentist pull your teeth if you don't behave yourself." Dentists should never tell a child that an operation will not hurt. There is nothing so repugnant to a child as a lie, I think it might be well, perhaps, to have a carefully prepared article written on this subject. The personal instruction of the dentist can do much. I remember one case where I convinced a mother of the necessity of caring for her child's teeth by instructing her from an anatomical specimen I had in my office, and so thoroughly convinced was she that she would not rest until she brought her husband and had him convinced also. I don't think we should extract the temporary teeth very often. The child should be trained to use the tooth brush as soon as possible, and if they want some dentifrice, as they frequently do, give them something simple and harmless, and it will do them good.

Dr. Goddard—Dr. Smith said in his address that money making is a science. Now I fancy that this suit of five rooms, this play house in one of them and this petting the children is only done in order to get them as patients. And that is all right. "Train up a child in the way it should go, and when it is old it will not depart therefrom."

Now I think about seven years ago I heard Dr. Redman say that when a six year molar was decayed he pulled it out, and if the one on the other side was sound he pulled that out too, and I want to ask him if he still adopts that course?

Dr. Redman—you never heard me say that. I did say and say yet, that I would not attempt to save a six year molar in a child under ten years of age, nor would I attempt to save a six year molar that ached, but then I only extract the one that aches and not the other.

The President—I have very much condemned the practice of not attempting to save these teeth under ten years of age. I claim that in every case where you can save a pulp in an adult you can do the same thing for a child, and if aching is caused by pressure upon the pulp and not by local trouble, it is just as reasonable to excavate that tooth, treat it carefully and cap it, as it would be in the tooth of an adult. If the pulp has been destroyed it is probably better to extract the tooth, to allow plenty of room for the wisdom teeth to come in and take the place of a good molar. But I always save them unless the pulps are dead.

Dr. Taft said he wished the nomenclature could be changed from six year old molar to first permanent molar. He would always preserve these teeth if possible, and thought tin or some such substance, better than gold for filling them, and soft gold better than cohesive foil. Had used Hill's stopping os-artificial, etc.

Dr. Kidd thought that many of these teeth could not be filled with soft foil, and he had sometimes used amalgam.

Dr. Redman said he had put in very large gold fillings in in the teeth of children between six and seven years of age, malleting them as hard as in adults and never saw any injurious results whatever. He thought a soft linen napkin worth all the brushes in the world—never recommended the use of a brush. Instead of floss silk, Coats' flax would answer quite as well, and he always gave a small spool of it to his patients before they left his office.

Dr. Edwards did not favor the public prints as a means of education on this subject because it would look like quackery.

Dr. Peabody said he had found bromide of potassium rubbed on the gums the most effectual remedy in relieving the pains of infants when cutting teeth. Subject passed.

The Society now made a final adjournment

